# Deformity correction and extremity lengthening in the lower leg: comparison of clinical outcomes with two external surgical procedures

**DOI:** 10.1186/s40064-016-3666-3

**Published:** 2016-11-24

**Authors:** Eugen Reitenbach, Robert Rödl, Georg Gosheger, Björn Vogt, Frank Schiedel

**Affiliations:** 1 Department of Orthopaedics, Klinikum Dortmund GmbH, Klinikzentrum Mitte, Dortmund, Germany; 2 Department of General Orthopaedics and Tumor Orthopaedics, Münster University Hospital, Münster, Germany; 3 Department of Pediatric Orthopaedics, Deformity Correction and Foot Surgery, Münster University Hospital, Münster, Germany; 4 Clemenshospital Muenster, Dept. of Pediatric Orthopedics and Deformity Correction, Muenster, Germany

**Keywords:** Deformity correction, Extremity lengthening, Ilizarov ring fixator, Taylor Spatial Frame

## Abstract

**Objective:**

Distraction osteogenesis is a method of stimulating the growth of new bone tissue in order to lengthen the extremities or bridge resected bone defects. In addition to the now-established intramedullary procedures, two different fixator systems are in use. The present study investigated the classical Ilizarov ring fixator (IRF) and a hexapod to assess the precision of lower-leg lengthening and complications classified using the Paley criteria for problems, obstacles, and complications. The study also examined the follow-up results in functional tests to assess outcomes in terms of range of motion in adjacent joints, daily activities, and quality of life.

**Patients and methods:**

A total of 43 patients (53 segments) who were treated over a period of 16 years were re-assessed. In 33 segments, treatment was carried out with the hexapod Taylor Spatial Frame (TSF); the conventional IRF was used in 20 segments. The patients’ mean age was 13.5 years (range 2–54 years). The follow-up examinations were carried out 2–15 years postoperatively and comprised measurement of a current leg axis view with the patient standing, calculation of a knee score, activity scores, ankle joint scores, and assessment of motor function and sensory function using appropriate scores in the lower leg and foot. The post-treatment health-related quality of life was assessed using the Short-Form Health Survey-36 questionnaire.

**Results:**

Using the Paley criteria, far fewer problems occurred in the TSF group in comparison with the IRF (TSF 12.1%, IRF 50%). In the problems category, significant differences were observed with regard to axial deviation (TSF 0%, IRF 36.8%) and pin infections (TSF 9.1%, IRF 40%). Comparison of the obstacles and complications did not identify any significant differences between the two groups. Analysis of the scores for the knee, activity, and motor function/sensory function also did not show any marked discrepancies, apart from a major difference in mobility in the upper and lower ankle joints with poorer findings in the TSF group.

**Conclusions:**

During treatment, the TSF ring fixator leads to fewer problems, fewer secondary axial translations, and fewer pin infections. However, with temporary transfixation of the ankle joints, the TSF system is also associated with postoperative deterioration in mobility in the upper and lower ankle joint.

## Background

In western Europe and North America, fixators have been in use for correcting and lengthening the extremities for around 35 years. Literature reports on the clinical results, range of complications, and surgical options also include larger cohorts of patients (Cattaneo et al. 1992; Ilizarov 1989a, b; Kristiansen et al. 2006; Paley 1988, 1990; Paley et al. 1989). The history of fixator devices goes back to the early twentieth century (Codivilla 1994; Wagner 1978; Wasserstein 1990; Wiedemann 1996) and was also decisively influenced by the publications of G.A. Ilizarov (Simard et al. 1992), which long remained inaccessible in the West. Ilizarov used bracing wires to attach bone fragments to rings, which were joined together using threaded rods, in order to apply targeted compression to the fracture cleft. In the process, rotation of the screws in the wrong direction accidentally led to distraction instead of compression, and mineralization of the distraction cleft was noted radiographically (Simard et al. 1992; Aronson 1994). The formation of new bone in a process known as distraction osteogenesis, through targeted traction on fractured or osteotomized ends, became known all over the world as the “tension–stress effect” in the decades that followed (Ilizarov 1989a, b). It forms the basis for what is known as callotasis in lengthening osteotomies using both external and also intramedullary lengthening systems. The term “callotasis” was coined by De Bastiani et al., who carried out lengthening procedures using fixators placed unilaterally along the bone. Since the late 1990s, hexapod fixator systems derived from the field of robotics have increasingly been used (Rödl et al. 2003; Seide et al. 1999).

Once Ilizarov’s work become more widely known, distraction osteogenesis was extensively tested in animal experiments and was investigated histologically and radiographically in humans (Ilizarov 1989a, b; Aronson 1994; Aronson et al. 1989; Delloye et al. 1990; Guichet et al. 1998; Kojimoto et al. 1988; Shearer et al. 1992). The following factors during the entire course of treatment (with fixation, distraction, and consolidation phases) emerged as being extremely important for ensuring adequate neocorticalization, satisfactory postoperative soft-tissue conditions, and good function in the adjacent joints: the greatest possible intraoperative protection of the periosteum and bone marrow, even during the osteotomy; stability of the fixator; an appropriate distraction rate and distraction frequency; and full weight-bearing (Ilizarov 1989a, b; Paley 1988; Aronson 1994; Aronson et al. 1989; Delloye et al. 1990; Kojimoto et al. 1988). The traction applied at the distraction cleft stimulates osteogenetic cells that accumulate on collagen fibers that are formed from a radiolucent fibrous interzone (FIZ). New vessels arise proximal and distal to the distraction cleft, and woven bone and bundle bone initially form around them before then being remodeled into finished lamellar bone. The most important characteristic of distraction osteogenesis is the absence (Ilizarov 1989a; Shearer et al. 1992) of the “intermediate cartilaginous phase” that would take place during enchondral ossification in fracture healing. This indicates that desmoid ossification (Ilizarov 1989a, b; Aronson 1994; Aronson et al. 1989) takes place during distraction, as in an epiphysial plate.

There are substantial differences in the construction of the three external fixators that were compared in the present study—the Taylor spatial frame (TSF) and the Ilizarov ring fixator (IRF).

The IRF (Ilizarov 1989a, b) consists of two rings that are attached to the bone by two orthogonally arranged full pins (with K-wires completely traversing the bone) and four telescopic rods. The pins are tensed to 90–130 kg using a special appliance; this increases the stability of the construct, as the tensed K-wires have a self-reinforcing effect if bending occurs and have a rigidity equivalent to that of 4-mm Schanz pins (Cattaneo et al. 1992; Aronson 1994; Aronson et al. 1989). The traction is applied concentrically, in contrast to the MLF (which is eccentric), ensuring consistent formation of regeneration tissue. In addition, the rotational rigidity and the curvature of the osteotomy ends prevent the development of malpositioning, while the axial elasticity promotes osseous regeneration (Paley 1988). The small pin diameter prevents the development of osteolysis and osteitis (Paley 1988; Aronson 1994; Aronson et al. 1989). This fixator has considerable disadvantages due to its bulky frame and limitations on everyday mobility (Donnan et al. 2003). As the assembly of the IRF appears initially easy to grasp, the comparatively shallow learning curve for the surgeon often only emerges as a result of complications during follow-up treatment of the first patients (Feldman et al. 2003).

The hexapod, introduced 15 years ago, was a novelty in medical technology that had its origins in the field of robotics. It was developed in the search for a suitable platform for simulating helicopter flights and is now widely used in technology—e.g., for aligning precision microscopy tables and satellite receiver dishes (Rödl et al. 2003; Seide et al. 1999). Its frame structure is based on the IRF, and the rings are also fixed to the bone using orthogonally arranged tensed wires and bone screws (half-pins). The first fixator of this type that became commercially available, the Taylor Spatial Frame (TSF^®^), is a frame based on the ring fixator that has a characteristic arrangement of six positioning braces that allow simultaneous correction of two bone fragments with all six degrees of freedom (Seide et al. 1999).

Following intraoperative assembly, “mounting parameters” such as the distance between the rings, positioning of the rings relative to the bone fragment, and the length of the braces are evaluated using web-based software, and a distraction plan is established using the data in which the braces are regarded as vectors, reducing the fixator to what it actually ought to be—a positioning aid for spatially redirecting bone fragments that is as free of complications as possible and provides a static holding system until bony consolidation takes place. As the deformity analysis and correction planning with this instrument were for the first time necessarily software-dependent, the learning curve relative to postoperative “surprises” should be much steeper in comparison with the IRF (Feldman et al. 2003).

The aim of the present study was to use the Paley classification (Paley 1990) to compare various fixator systems for lengthening the lower leg, with simultaneous deformity correction playing at best a subordinate role. In addition, despite heterogeneous treatment data and historically widely differing numbers of patients, the aim was to obtain fundamental information about the long-term follow-up. The focus was on two questions: the aspects of functionality (range of motion and daily activity) and of quality of life (quality of life and psychosocial health) in everyday life, relative to the type of fixator used (Paley et al. 1989).

## Patients and methods

In lengthening osteotomies carried out from 1992 to 2008 at our university center, a total of 95 patients received treatment in the lower leg using two different external fixators—the Taylor Spatial Frame (TSF) and the classic Ilizarov ring fixator (IRF). The criteria for inclusion in the study consisted of distraction by at least 20 mm without simultaneous varus or valgus osteotomies of more than 10° at the tibia. Patients with less than 20 mm of planned lengthening and more than 10° of planned axial adjustment were excluded in order to rule out cases mainly involving only gradual adjustment. The rate of patients who were lost to follow-up was 48%. Due to the long periods between the operation and the follow-up examination, changes of residence meant that only limited tracing of patients was possible—particularly those who were treated in the 1990s. Although it is compulsory in Germany for residents to be registered at Residents’ Registration Offices, no inquiries for official assistance in tracing patients were made of these offices, due to the high costs expected to result after multiple changes of residence. The number of patients in the follow-up is 43, with 53 lengthened segments. Thirty-three segments were treated with the TSF, 20 with the IRF. The patients’ mean age at the time of surgery was 13.5 years (range 2–54 years). There were 14 female patients (24 segments) and 19 male patients (29 segments). The follow-up examinations took place over periods of 2–15 years postoperatively, between the fall of 2010 and 2012. A distinction was made between acquired and congenital entities. Monofocal and bifocal osteotomies were carried out in all of the groups. There were 13 monofocal and 20 bifocal osteotomies in the TSF group, eight monofocal and 12 bifocal osteotomies in the IRF group. With monofocal osteotomies, no distinction was made in this study between proximal and distal osteotomy. The mean preoperative leg length difference in all groups taken together was 56.25 mm, with 53.67 mm in the TSF group, 58.8 mm in the IRF group.

The leg length differences and centre of rotation and angulation (CORA) were measured preoperatively by taking leg axis views with the patient standing and corresponding lateral radiographs (Rödl et al. 2003; Seide et al. 1999; Feldman et al. 2003). In addition, the distal medial/lateral femoral angle, the proximal medial/lateral tibial angle, the distal tibial angle and the mechanical axis deviation (MAD) were measured. With TSF, preoperative planning included establishment of the reference fragment (Feldman et al. 2003). The position of the tibia relative to the reference ring (Seide et al. 1999) was used to define the mounting parameters.

With the IRF distraction was increased at 1 mm/d and with tight radiographic check-ups. The mean period in days to the start of distraction was 10 days in all of the groups. Fortnightly follow-up checks with a clinical examination and radiography were carried out, and leg axis views with the patient standing were taken every 3 months to assess the regenerate bone and to measure the articular angle and leg length difference in each patient.

With approval from the hospital’s review board and with written informed consent from the patients before the follow-up examination, a standardized questionnaire assessing the postoperative physical and mental state of health was sent to each patient in advance of the reassessment. The Short-Form Health Survey-36 (SF-36) questionnaire was used, with an evaluation of 36 questions consisting of a total of eight components and statements about patients’ perception of their physical and mental health. The questionnaire’s eight categories take into account physical functioning, physical role functioning, bodily pain, general health perceptions, vitality, social functioning, emotional role functioning, and mental health. A scoring scheme for the individual categories can be used to provide a physical summary score and a mental summary score, allowing a subjective assessment of the patient’s state of health. The results are standardized into an integral score with points from 0 to 100, with a high score representing a better subjectively perceived overall state of health.

The follow-up comprised an examination in accordance with various objective scores for each joint. For the knee joint, the Knee Society score as described by Insall et al. (1989) was used, which is also divided into an objective clinical section (the knee score) and a subjective functional section (the function score). The knee score takes into account the aspects of pain, range of motion in degrees, and ligament stability, with 0–100 points being possible.

The Tegner activity score (Tegner and Lysholm 1985) is used to assess postoperative physical activity. Assessment of the upper ankle joint (UAJ) and lower ankle joint (LAJ) was objectivized using the Weber score as an instrument. It features six categories, each including the three subjective dimensions of pain, walking distance, and activity at work and the three objective dimensions of radiographic diagnosis and clinical examination of the UAJ and LAJ using the neutral-0 method. The value 0 indicates the best possible result. Motor function in the extremity during plantar flexion, dorsal flexion, inversion, and supination is assessed using the Motor Score, divided into six levels of 0–5 points. The neurological status comprised the Neuropathy Symptom Score (NSS) and Neuropathy Deficit Score (NDS). The NSS covers the type of symptoms (stinging, numbness, paresthesias, faint feeling, cramp, pain), the location of the symptoms (foot, lower leg), exacerbation depending on the time of day and symptomatic improvement depending on exertion. The NDS assesses reflex status in the Achilles tendon, vibration sensitivity at the metacarpophalangeal joint of the great toe, pain on the back of the foot, and temperature sensitivity in both feet.

After the follow-up examination, all of the examination results obtained during and after completion of the fixator wearing period, as well as the current results, were correlated with the Paley criteria for problems, obstacles, and complications (Paley 1990). Problems in this context consist of difficulties during the distraction phase or consolidation phase that can be managed conservatively up to the time of fixator removal. Obstacles are events requiring secondary surgical intervention or an intervention under anesthesia, or an unplanned additional hospitalization lasting more than 24 h, which are resolved by the time the treatment is completed. Complications are defined as difficulties that it has not been possible to correct before removal of the fixator, although all possible conservative and surgical measures aimed at correcting them have been exhausted. An additional distinction is made between mild complications followed by successful conservative treatment after fixator removal and severe complications with persistent symptoms or surgery being required or with complete failure to achieve the goal of treatment (e.g., amputation, persistent leg length differences, limited everyday mobility).

All of the data were recorded using Microsoft Office Excel 2010 and were processed using descriptive statistics with IBM SPSS Statistics, version 21.0. For statistical calculations, the normal distribution of the groups was checked using the Kolmogorov–Smirnov test. If a normal distribution was present, the *t* test was then used to calculate significance with a confidence interval of 95%. When the variables were not normally distributed, the Mann–Whitney test for comparisons between two groups was used.

## Results

The postoperative follow-up period was 2–15 years. In all 43 patients, their medical history was noted, a leg axis view with the patient standing was taken, a physical examination was carried out in accordance with the relevant scores, and a questionnaire was completed to assess the patient’s subjective physical and mental state of health. The significance level was set at *P* < 0.05 in this study. The relevant ranges for the parameters analyzed are shown in Table 1. The mean leg length difference was 56.25 mm (range 0–170 mm) for all groups, with means of 53.7 mm in the TSF group, 58.8 mm in the IRF group. The distraction plan was achieved in a total of 41 segments (77.3%), 26 of which (78.8%) were in the TSF group and 15 (75%) in the IRF group. The mean lengthening distance was 62.35 mm (range 20–240 mm) overall, with 59.7 mm in the TSF group and 65 mm in the IRF group. The mean fixator wearing time was 219.8 days (range 76–514 days) in all groups, with 192.1 days in the TSF group and 247.4 days in the IRF group. There were no significant differences, despite the much shorter wearing time in the TSF group in comparison with the other groups.

**Table 1:** Parameters investigated

	TSF	IRF
N	33	20
Sex, f/m	14/19	10/10
Etiology: congenital/acquired	25/8	11/9
Age at surgery, years (range)	14.7 (2–54)	12.2 (7–20)
Secondary interventions	25	14
Operated leg, right/left	15/18	13/7
Preop. LLD in mm, mean (range)	53.7 (0–170)	58.8 (0–140)
Lengthening in mm, mean (range)	59.7 (20–240)	65 (30–140)
Successful correction	26	15
Postop. LLD in mm, mean (range)	15.3 (0–77)	21.4 (0–45)
Amputations	2	2
Osteotomy type, monofocal/bifocal	13/20	8/12
Distraction period, days (range)	68.7 (24–394)	88.3 (36–361)
Fixator wearing period, days (range)	192.1 (76–456)	247.4 (111–514)
Lengthening speed, mm	1.1	0.9
Healing Index, months/cm (range)	0.39 (0.17–1.31)	0.44 (0.2–1)
Distraction Index, days/cm (range)	11.6 (5.1 –39.4)	13.2 (6–30.1)
Total Knee Score, mean (range)	85.4 (60–100)	83.9 (60–100)
Knee Function Score, mean (range)	80.4 (5–100)	78 (30–100)
Tegner Activity Score, mean (range)	3.6 (1–9)	3.4 (2–6)
UAJ/LAJ Weber score, mean (range)	9.8 (0–20)	6.7 (1–20)
Motor Score, foot	4.1	4.3
Neuropathy Sensitivity Score	1.9	1.8
Neuropathy Deficit Score	0.52	0.35

*IRF* Ilizarov ring fixator, *LAJ* lower ankle joint, *LLD* leg length discrepancy, *TSF* Taylor Spatial Frame, *UAJ* upper ankle joint

During the course of treatment, lower leg amputations were carried out in three patients and a Chopart amputation in one patient; two of these patients were in the TSF group and two in the IRF group.

The mean postoperative leg length difference was 18.35 mm (range 0–77 mm) in all groups, with 15.3 mm in the TSF group and 21.4 mm in the IRF group, A significant difference was noted in the IRF group, with a higher mean postoperative leg length difference in comparison with the MLF group.

During the course of treatment, a total of 39 secondary interventions were required, 25 of which were in the TSF group and 14 in the IRF group. The mean distraction period was 78.5 days (range 24–394) overall, with means of 68.7 days in the TSF group and 88.3 days in the IRF group. It was significantly shorter in the TSF group than in the IRF group. The Healing Index—the period in months needed to achieve a lengthening of 1 cm—showed a mean of 0.42 (range 0.17–1.31) in all groups. The parameter used to calculate the achievement of 1 cm of lengthening in days, known as the Distraction Index, showed a mean of 12.4 days (range 5.1–39.4 days) in the two groups. No significant differences between the groups were noted with regard to the Healing Index or Distraction Index.

The mean Knee Society Score was 84.65 (range 60–100) in all groups, with 85.4 in the TSF group and 83.9 in the IRF group. The Knee Function Score was used as a parameter for assessing subjective function and showed a mean of 79.2 (range 5–100) in all groups, with 80.4 in the TSF group and 78.0 in the IRF group. Physical exertion during leisure time and at work was assessed using the Tegner Activity Score, which showed a mean of 3.5 points (range 1–9) for all groups, with 3.6 in the TSF group and 3.4 in the IRF group. No significant discrepancies between the three groups were noted in any of the above three parameters.

The UAJ and LAJ were also analyzed using the Weber score, which showed a mean of 8.25 points (range 0–20) in all two groups, with 9.75 in the TSF group and 6.7 in the IRF group. There were significantly lower scores in the TSF segments in comparison with the IRF group. Finally, the Motor Score was used to assess motor function, and the Neuropathy Symptom Score and Neuropathy Deficit Score were used to assess the lower leg and foot.

In the analysis of complications occurring during treatment in accordance with the Paley criteria (problems, obstacles, and complications; Table 2), a significant difference was noted with regard to the overall number of problems, which were much lower in the TSF group than in the IRF group. A significant difference was also observed in the occurrence of axial deviations in the “problems” subgroup, with the TSF segments showing no axial malpositioning at all in comparison with the IRF group. There were also no significant differences with regard to pin problems, although the rate in the TSF group was much lower than that in the IRF group. None of the other parameters relating to the Paley criteria showed any significant differences (Table 2).

**Table 2:** Paley criteria

Parameter	TSF			IRF			Total
	Problems	Obstacles	Complications	Problems	Obstacles	Complications	
Muscle contraction	1	5	0	0	2	0	8
Joint dislocation	0	2	0	0	1	0	3
Axial deviation	0	7	0	7	2	1	17
Neurological deficit	0	0	0	0	1	0	1
Vascular complications	1	0	0	0	0	0	1
Premature consolidation	0	3	0	0	0	0	3
Delayed consolidation	0	2	0	1	0	0	3
Pin problems	3	1	1	8	2	0	15
Repeat fracture	0	2	1	0	3	1	7
Joint stiffness	0	2	0	0	0	0	2
Other	0	2	1	0	2	1	6
Total	5	26	3	16	13	3	66

*IRF* Ilizarov ring fixator, *TSF* Taylor Spatial Frame

**Fig. 1:**
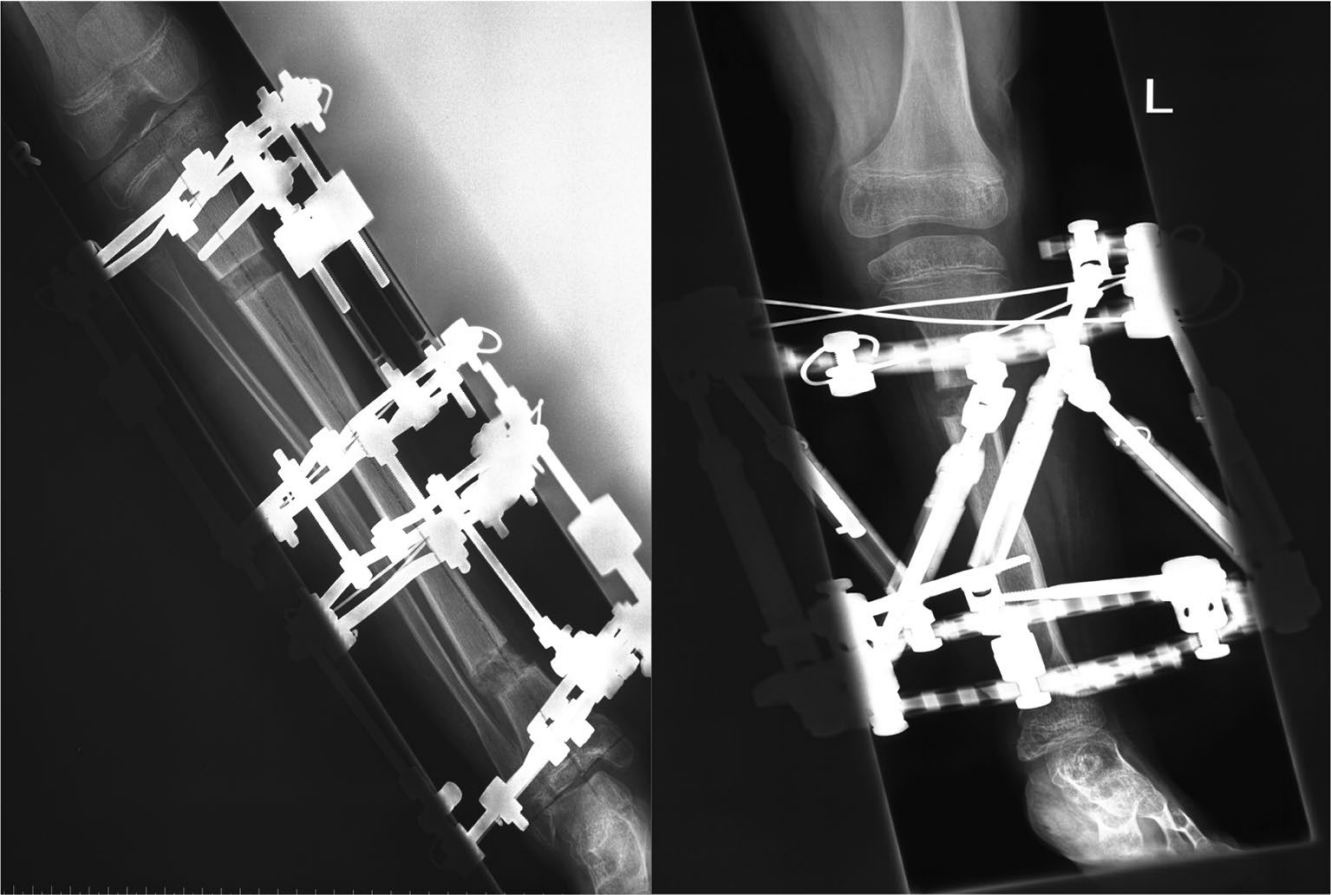
Typical assembly of the external fixators studied:, Ilizarov ring fixator (*left*) and Taylor Spatial Frame (*right*)

Comparison of the SF-36 data collected showed a mean physical summary score of 43.15 points (range 24–59), with a mean of 42.18 (range 25–59) in the TSF group and mean of 44.05 (range 24–56) in the IRF group. The figures did not show any significant associations between the fixator procedures and the patients’ subjective state of health. Significant differences were noted in the individual subscales (Table 3) for physical function, physical role function, and social function in favor of the TSF group, with a much lower mean in comparison with the IRF group. The points for the eight individual components are listed in Table 3; the values are based on standard data for Germany (Fig. 1).

**Table 3:** Analysis of the Short-Form Health Survey-36 (SF-36) subscales

Fixator type	Physical summary scale	Mental summary scale	Physical function	Physical role function	Pain	General health perception	Vitality	Social function	Emotional role function	Mental health
*TSF*										
Mean	42.2	51.1	56.7	56.1	70.4	73.8	62.3	73.9	85.9	76.2
n	33.0	33.0	33.0	33.0	33.0	33.0	33.0	33.0	33.0	33.0
SD	9.1	8.8	24.8	40.5	23.6	14.4	15.6	27.1	34.4	12.0
Min.	25.0	20.0	5.0	0.0	31.0	35.0	25.0	0.0	0.0	36.0
Max.	59.0	63.0	95.0	100.0	100.0	92.0	85.0	100.0	100.0	96.0
*IRF*										
Mean	44.1	53.2	67.0	87.5	67.7	61.3	59.8	88.1	98.3	75.6
n	20.0	20.0	20.0	20.0	20.0	20.0	20.0	20.0	20.0	20.0
SD	9.5	5.6	26.9	25.0	27.9	23.8	18.9	17.0	7.4	14.7
Min.	24.0	43.0	20.0	25.0	22.0	25.0	15.0	50.0	66.7	44.0
Max.	56.0	61.0	100.0	100.0	100.0	92.0	80.0	100.0	100.0	96.0

*IRF* Ilizarov ring fixator, *Max*. maximum, *Min.* minimum, *SD* standard deviation, *TSF* Taylor Spatial Frame

## Discussion

The results of this study allow the outcomes with two different external fixation procedures for lengthening the lower leg to be compared in relation to the associated complication rates based on the Paley criteria. The study assesses the precision of the results achieved with the planned length adjustments and allows comparison of joint scores at the follow-up examination, taking into account the patients’ physical and mental state of health, using standardized outcome measurement tools.

Comparison of the three procedures showed that there were far fewer problems in the TSF group (n = 5/33, 15%) than in the IRF group (n = 15/20, 75%). Axial deviations were not observed in the TSF group, but were frequent in the IRF group (n = 7/20, 35%). Pin problems were rarer in the TSF group (n = 3/33, 9%) than in the IRF group (n = 8/20, 40%). Comparisons of obstacles and complications in accordance with the Paley classification did not show any significant differences.

As earlier studies have already reported, the fact that the TSF fixator shows better results in relation to problems—i.e., conservatively resolved cases—is attributable to its high level of flexibility for correction in all six degrees of freedom, with no need for fixator modification.

The ratio of monofocal to bifocal osteotomies in the two types of ring fixator, TSF (n = 13/20) and IRF (n = 8/12), and the mean lengthening in these subgroups with bifocal lengthening (TSF 60 mm, IRF 65 mm) is almost equal. However, there was a marked difference in the fixator wearing period in days between these two subgroups. In the TSF group with bifocal lengthening procedures, the figure was 192 days, while in the IRF group it was 247.4 days, with distraction periods of 69 days in the TSF group and 88 days in the IRF group. The most likely explanation for this is the support for planning provided by the software.

When hexapods were first introduced in the late 1990s, new discoveries were made in relation to deformity correction in the extremities, as the involvement of technology and correction processes made more intensive training necessary for the users in analyzing deformities and in planning the corrections. The findings of the present study can therefore only be generalized to a limited extent, partly because of the heterogeneous distribution of patients among the three types of fixator. The two ring fixators have previously been compared several times with regard to various aspects such as complication rates, precision, and mechanism (Paley 1988; Paley et al. 1989; Rödl et al. 2003; Seide et al. 1999; Manner et al. 2007; Matsubara et al. 2006). The results have generally shown that hexapods were associated with lower complication rates and greater precision in the correction procedure and consequently in the results. However, this needs to be qualified by emphasizing that secondary corrections and modifications with the Ilizarov fixator were often carried out with anesthesia or on an in-patient basis, so that the difficulty was classified as an obstacle in the Paley classification—whereas changes in the hexapod protocol only represent a problem whose solution does not require any further anesthesia or hospitalization.

Interestingly, the results relating to function (range of motion and daily activity), quality of life, and psychosocial state in daily life relative to the type of fixator used show that hexapods still do not solve problems involving upper ankle joint mobility and talipes problems.

Markedly poorer values on the Weber score were observed in the TSF group. The parameters for which data were collected at the follow-up examination do not show any clear differences between the three procedures, with the exception of the Weber UAJ/LAJ score. Surprisingly, the Weber score in the TSF group, with a mean of 9.8 points, was poorer than in the other group. In comparison, the mean scores were 6.7 in the IRF group. One possible reason for the limited mobility in the UAJ might be the cross-joint foot fixture used to prevent talipes and UAJ subluxation during the distraction phase. In contrast to the monolateral fixators, in which technically easy temporary arthrodesis is not possible in the fixator, this was done increasingly often with the Ilizarov ring fixator (n = 13/20 patients) and in almost all of the patients treated with lower-leg TSF (n = 27/30). Foot fixture is usually used to stabilize the UAJ in patients with fibular aplasia, those with hemimelia with an unstable UAJ, or when talipes is already present. As regular UAJ exercise with physiotherapy is not possible during temporary transfixation, restoration of the original UAJ mobility following the completion of fixator treatment is much slower, as is probably reflected in the below-average score particularly in this treatment group, which is chronologically the youngest. Most of the patients in the TSF group were children with the congenital conditions mentioned earlier. This problem was also taken into consideration at the time, and distraction at the UAJ following assembly was usually set to at least 1 cm in order to protect the cartilage during the transfixation months. This problem needs to be addressed in the future; whenever possible, surgical transfixing of the joints should be avoided through consistent physiotherapy and use of other aids—or if appropriate, early selective disassembly of the foot fixture after the end of distraction should be considered. The UAJ adjusting screws familiar from lengthening procedures using medullary nails might also play a role for fixator patients in the future (Belthur et al. 2008).

## Conclusions

In summary, the TSF fixator continues to represent an innovative further development for deformity correction in comparison with the IRF. Its software-based planning and the flexibility of correction it provides, with several degrees of freedom, markedly reduce the rates of problems occurring during the distraction phase and thus shorten both treatment times and fixator wearing times, while still achieving the correction target. The system, which has been available for over 15 years, has probably made a substantial contribution to surgeons’ awareness of these matters and of the need for precise deformity analysis and correction planning.
